# Finding and Characterising Active Slip Systems: A Short Review and Tutorial with Automation Tools

**DOI:** 10.3390/ma14020407

**Published:** 2021-01-15

**Authors:** James S. K.-L. Gibson, Risheng Pei, Martin Heller, Setareh Medghalchi, Wei Luo, Sandra Korte-Kerzel

**Affiliations:** Institute for Physical Metallurgy and Materials Physics, RWTH Aachen University, 52056 Aachen, Germany; pei@imm.rwth-aachen.de (R.P.); heller@imm.rwth-aachen.de (M.H.); medghalchi@imm.rwth-aachen.de (S.M.); luo@imm.rwth-aachen.de (W.L.); korte-kerzel@imm.rwth-aachen.de (S.K.-K.)

**Keywords:** micromechanics, nanoindentation, micropillar compression, slip systems, intermetallics

## Abstract

The behaviour of many materials is strongly influenced by the mechanical properties of hard phases, present either from deliberate introduction for reinforcement or as deleterious precipitates. While it is, therefore, self-evident that these phases should be studied, the ability to do so—particularly their plasticity—is hindered by their small sizes and lack of bulk ductility at room temperature. Many researchers have, therefore, turned to small-scale testing in order to suppress brittle fracture and study the deformation mechanisms of complex crystal structures. To characterise the plasticity of a hard and potentially anisotropic crystal, several steps and different nanomechanical testing techniques are involved, in particular nanoindentation and microcompression. The mechanical data can only be interpreted based on imaging and orientation measurements by electron microscopy. Here, we provide a tutorial to guide the collection, analysis, and interpretation of data on plasticity in hard crystals. We provide code collated in our group to help new researchers to analyse their data efficiently from the start. As part of the tutorial, we show how the slip systems and deformation mechanisms in intermetallics such as the Fe_7_Mo_6_ μ-phase are discovered, where the large and complex crystal structure precludes determining a priori even the slip planes in these phases. By comparison with other works in the literature, we also aim to identify “best practises” for researchers throughout to aid in the application of the methods to other materials systems.

## 1. Introduction

In the knowledge-based development of structural materials, we commonly design and manipulate their internal (micro)structure at several length scales to control the physical mechanisms governing deformation. While the origin of mechanical strength, toughness and creep resistance is well studied in most engineering metals, only very little is known about the properties of more complex crystal structures, despite their wide use as reinforcement phases and the undiscovered potential in their sheer number and variability [1,2,3,4]. To find and design new materials, we need to close this gap in knowledge by revealing the physical deformation mechanisms and resulting mechanical properties in these complex phases. This ranges from the understanding of a handful of defects affecting functional properties in semiconductors, via the mechanical properties of the many binary and ternary intermetallic precipitation phases that develop intentionally or on their own accord in modern alloys [1,5,6,7], to finally the performance of the many compounds studied as hard coating materials. Several gaps also remain in our current understanding with respect to long-established metals and their alloys. In these cases, fundamental studies of plasticity may be concerned, for example, with body-centred cubic (BCC) crystals and their more numerous slip systems [8,9,10] or the interaction of lattice defects, such as dislocations and their associated stacking faults [11,12] or the pile-up and transmission of dislocation at grain and phase boundaries [8,13,14].

Here, we will focus on single crystalline BCC [8] and intermetallic crystals [15,16] as examples, but the methods, analyses and code can be applied to any material that forms sufficiently clear traces of plasticity on its surface and in which these traces are governed by the crystal structure.

With respect to the intermetallics taken as an example here [15,16], there are not only a great number that remain to be studied, but many different applications in which their mechanical properties need to be known beyond a simple stiffness or hardness. They are used as phases for alloy reinforcement [1], they form unintentionally in modern materials with high alloying contents, such as the superalloys [17,18], and for the optimistic researcher they may still be hiding a new class of bulk alloys amongst their numbers that combines safety as a structural material with great resistance to harsh environmental conditions.

The study of intermetallic phases is a steadily increasing field of research, with over 3350 papers published on this topic in 2019, according to a Web of Science search for the topic. Research on their mechanical properties is, however, far less prominent, comprising on average less than half of this body of work. This contrast comes in part from several experimental challenges: (i) due to the complex crystal structure of these phases, they typically show very limited room-temperature plasticity [5,19,20]; (ii) as a direct result of the poor ductility, these phases are only tolerated either as small precipitates, or introduced as small reinforcing phases, such that the properties of the parent alloy are not significantly reduced [2,21]. This small size consequently limits the tests that can be performed; (iii) finally, as only small amounts of these phases are formed or used, it is difficult for the researcher to even obtain experimentally useful volumes of known chemical composition and with known thermomechanical history.

Similar limitations exist with respect to fundamental studies of plasticity in single or bicrystals of pure metals and alloys [22]. The draw-backs of small-scale testing, such as size effects on quantitative values and the danger of affecting dislocation nucleation, multiplication and interaction in a given setting [22,23,24], are commonly outweighed by the difficulty in preparing single crystals large enough for macroscopic testing, particularly of alloys. In the case of bicrystals, the difficulties are even more severe as the included grain boundary must be both well-defined and straight, and bi-phase samples with a single or few phase boundaries can most of the time not be synthesised at all.

These limitations are compensated for by the strengths of nanoindentation and micro-mechanical testing. These enable the investigation of small volumes of material [22,25,26,27], allowing intermetallic phases to be tested within the parent alloy or even small fragments of specially prepared alloys through arc melting, while boundaries or specific crystal orientations can be targeted straightforwardly in any metallic alloy or metallic-intermetallic composite. Perhaps the greatest strength of micro-mechanical testing, however, is the suppression of brittle fracture, even in uniaxial microcompression tests [4,28,29,30]. These subsequently allow the study of plastic properties essential for the performance of many modern engineering alloys in which a governing microstructural length is often at the same length scale of microcompression tests; that is, from 100 s of nm to a few µm.

The experimental space occupied by metallic alloys and intermetallic compounds is enormous, covering binary, ternary and subsequent systems, the resultant crystal structures and their orientation within the sample, local chemistry, impurities, and precipitations, and how deformation is shared across interfaces. In addition come the influences from the ‘traditional’ global variables: effects of temperature and strain rate [31,32]. It can be, therefore, safely assumed that, in the case of intermetallics in particular, no starting point exists for the characterisation of the vast majority of these phases, or at least many unknowns remain concerning their mechanical and particularly plastic behaviour.

The following questions, therefore, need answering for a new compound of interest:

1. What slip systems are operating in my crystal?
2. What are the critical stresses required to operate these slip systems?
3. What are the mechanisms by which plasticity propagates?

Answering these questions not only allows further academic studies towards knowledge-based materials research, but also provides the tools to improve and refine alloys using intermetallics in practise. The answers additionally help us expand our understanding of fundamental processes even in metallic alloys that have been studied already in great depth.

This text should serve as both a short review and a tutorial, outlining the basic methods and giving practical examples of uses and code that can be employed for those embarking on studies of crystal plasticity using nanomechanical testing.

## 2. Materials and Methods

**Figure 1:**
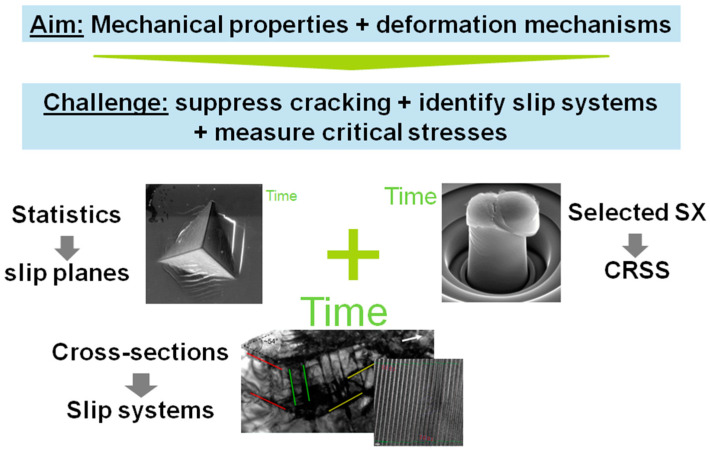
Typical combination of methods employed to unravel the mechanical properties and underlying deformation mechanisms in materials where these might not be fully understood or known at all. Their order and selection is normally based on the investigative depth, e.g., hardness versus a critical resolved stress on an individual slip system, time, and cost.

In the following, we outline all those techniques (in their typical order of application) that are useful in studying the mechanisms of plasticity in a crystal for which very little is known a priori, shown in Figure 1. The order of application is usually determined by cost and time, starting with a screening of possible slip planes and measurement of basic mechanical properties with indication of anisotropy, if present. This is usually accomplished by nanoindentation. For a detailed interpretation of the resulting surface traces of slip and orientation-dependence of properties, such as hardness and modulus, the crystal orientation of the material must be known at the location of each indent. This, in turn, is normally measured using electron backscatter diffraction (EBSD). Together, these techniques answer our first question detailed above. For the second, as indentation relies on a three-dimensional stress field, critical resolved shear stresses are out of reach (or can only be estimated from iterative modelling where the potential slip systems are already known). For this reason, microcompression is usually employed as a close proxy to conventional uniaxial testing. Further geometries are available, such as tensile testing, cantilever bending or shear experiments. However, all of the latter usually require the use of nanomechanical testing equipment inside an electron microscope or using a high-resolution surface imaging and positioning stage.

For this reason, we focus here on the studies accessible to those with the basic instrumentation of a scanning electron microscope (SEM) that can image down to the required scale of slip traces around indentations and perform orientation imaging by EBSD, i.e., the combination of nanoindentation, microcompression, SEM and EBSD. Ideally, such studies may result in final analyses—and an answer to the final question—by transmission electron microscopy (TEM) to confirm or find the underlying dislocation Burgers vectors and, therefore, complete slip system information for selected samples.

### 2.1. Sample Preparation

The preparation of a suitable sample for testing is the natural first step in this analysis, however this is not so straightforward at it may first seem. It must be remembered that the goal is to extract and quantify plasticity mechanisms, i.e., neither simply mechanical testing by nanoindentation nor an isolated analysis via electron microscopy, such as orientation imaging by EBSD. The sample preparation must therefore be suitable for both methods to work. While the end goal for both the microscopy and the indentation is a damage-free surface, the calculation of indenter contact area implicitly assumes a flat surface is being indented and any other nanomechanical test geometries (micropillars, microcantilevers, etc.) also require a flat contact surface at their top. As such, chemical polishing, i.e., electropolishing, is, in the authors’ experience, a poorly suited technique. Even chemo-mechanical polishing with colloidal silica in a mildly reactive suspension often over-polishes at grain or phase boundaries, leading to uneven samples. We found that polishing down to a sub-micron diamond finish followed, if necessary, by a short polish with colloidal silica for only a few minutes, produces the best results. Both particles, diamond and silica, must be washed off very carefully before proceeding to nanomechanical testing.

### 2.2. Nanoindentation

The goal of the indentation tests is to induce plastic deformation in the crystal. If slip is concentrated on individual planes and cross-slip is ideally limited, then a considerable portion of slip on these planes will intersect the polished surface of the specimen, producing a slip step. The angle this step makes with the surface, and the angle within the plane of the surface are characteristic of the operating slip plane. This first angle—that made with the surface—is subsequently discarded in the interest of speed in the following analysis, due to its measurement requiring a three-dimensional analysis technique such as atomic force microscopy (AFM) [33,34]. An increase in non-unique solutions results (by 72% for example in the µ-phase [16]), although the exact value will depend on crystal symmetry, as different potential slip planes may intersect the surface at the same angle. However, this type of analysis comes with the benefit that the indents can be rapidly analysed using SEM imaging and interpreted based on the local crystal orientation. TEM observations typically show that each visible slip step corresponds to significant dislocation activity on a single slip plane, rather than a tightly spaced group of planes with only a handful of dislocations [6]. However, if the reverse were true, the indentation tests nevertheless still allow an observation of which slip planes are activated in a complex crystal as a function of crystal orientation, in order to guide further microcompression tests.

Nanoindentation loads or depths should thus be chosen to be “just right”; namely, producing enough deformation such that clear slip lines are obtained without producing overlapping deformation bands on the surface, or significant deformation underneath the indent to preclude analysis by TEM. As an example, for the µ-phase in the Fe-Mo system [16], with an average hardness of 11.7 GPa, this corresponded to indents made to 100 mN and 200 mN, with an indentation depth in the range of 800–1000 nm. In most cases, even nanoindentation systems limited to smaller loads, e.g., 50 mN, can be used successfully to introduce deformation suitable for slip line analysis. Note that if a material forms continuous pile-ups, such as observed in most metals, there is, of course, no scope for a slip trace analysis in indentation (but it is also unlikely to yield any new insights as the simple metals that form these pile-ups are already well-studied materials). In contrast, many metals do show slip traces in microcompression and other small-scale geometries, so those methods might still be worth pursuing (see further below) and the initial step of identifying operative slip systems is then often obsolete as the active planes are well established in the literature.

### 2.3. Determination of Crystal Orientation

It is important that one has the knowledge of which grain orientations were indented, therefore EBSD mapping of the indented area is essential. Depending on the grain size of the investigated material, this can be done pre- or post-indentation. Mapping after the indents have been performed is experimentally more straightforward, as it is only performed where indentations were successful and they are then clearly visible in the orientation map as clusters of zero solutions, but the map quality will consequently be degraded in the deformed regions.

**Figure 2:**
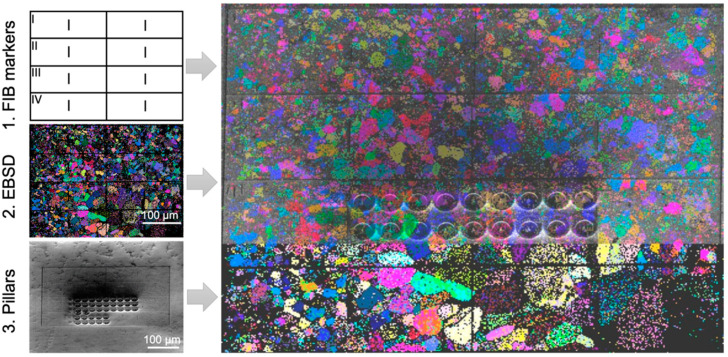
FIB-milled grid to enable correlation and alignment for nanomechanical testing in a Nb_2_Co_7_ intermetallic sample. The sample was arc-melted and subsequently heat treated for 1000 h at 1000 °C, followed by mechanical polishing and a final colloidal silica polishing step. Image reproduced in part from [36].

In order to confine deformation to a single grain, it is recommended that the grain size is significantly larger than the indent width, where, for example, indents can be spaced at least 10 µm away from the grain boundary [6]. However, given that nanoindentation studies have shown that values of indentation hardness are not significantly influenced by indents spaced >10 times the indentation depth (e.g., 1 µm deep indents should be at least 10 µm away from each other) [35], it is plausible that a similar criterion applies to grain boundaries. For samples where this is not possible and the grain size is close to the final indent width, or, more commonly, for samples where oxidation can be a problem and the EBSD scans should be conducted as quickly as possible, the maps should be correlated to robust microstructural features. When planning such markings, it should be remembered that secondary electron (SE) contrast can be very different to optical contrast, not to mention the vastly decreased resolution present in the optical microscopes of indenter systems. Therefore, large precipitates, dots of ink, a widely spaced indentation grid performed at high load or deliberately induced scratches from diamond scribes are all good options for this correlation. It is also possible to use a focused ion beam (FIB) to FIB-mill fiducial markers, or even a regular grid (Figure 2), provided milling currents are low enough that damage is confined only to roughly the first 100 nm. This gives a compromise between visibility under the microscope and zero solutions in the EBSD map. Milling to these depths then only damages regions where surface roughness and errors in tip calibration make indentation data difficult to extract in any case. It will, therefore, not interfere too much with deeper indents. Of course, the latter assumption should be verified for a given material, e.g., by comparison of similarly ion bombarded and pristine surfaces.

### 2.4. Measurement of Experimental Slip Lines

**Figure 3:**
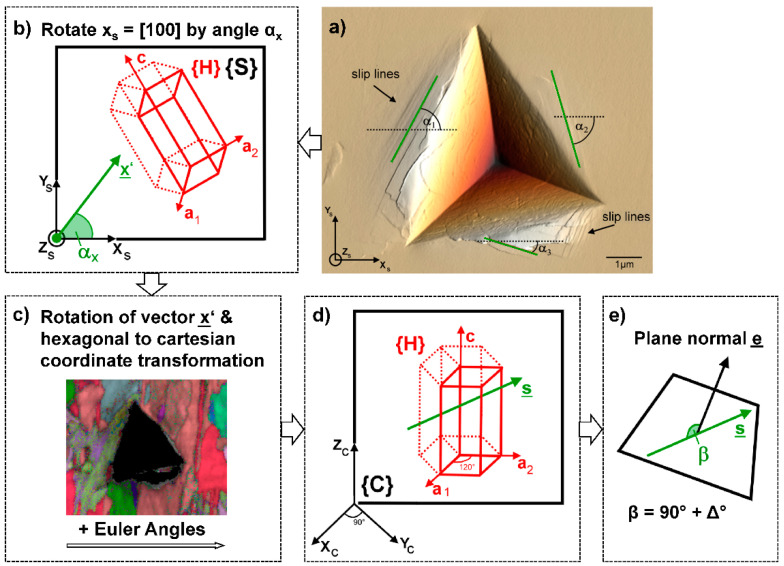
Schematic representation of the surface trace analysis: (**a**) angular measurement of traces. (**b**) rotation of sample image horizontal (*x*-axis) to obtain trace vector. (**c**) Further rotation by Euler matrix and hexagonal to cartesian transformation, show in (**d**) as the vector in crystal coordinates. (**e**) If the dot product of plane normal and vector equals 90° ± Δ the observed surface trace lies within plane. The indent in (**a**) is from a Fe_7_Mo_6_ µ-phase sample produced by arc melting, annealed for 1000 h at 800 °C and mechanically polished down to a colloidal silica finish. Image reproduced from [16].

The identification of slip planes around indents is not restricted to hard crystals, where it might be expected that the number of slip planes are limited. Indeed, this kind of analysis is also performed on metallic crystals (e.g., [37,38,39,40]). The analysis of the surface traces is well-described in the literature, e.g., [16], but is repeated in brief and illustrated here so as to simplify matters for the reader (Figure 3). The goal is to determine whether an observed surface line could belong to the intersection of a slip plane in the indented crystal with the surface of the sample. This yields two important pieces of information: the active slip planes (though without their slip directions) and their likely relative ranking in terms of critical resolved shear stress (CRSS). As detailed below, the observation of more slip planes than would be predicted from the crystal symmetry implies a low CRSS for the slip plane in question. Subsequent microcompression tests then need only target the existing slip planes, and the determined CRSS values can be cross-checked with the slip plane operation frequency.

The angle that the slip trace makes with a reference axis, such as the image horizontal, is measured in the sample reference system or ‘sample space’ (Figure 3a). The image horizontal is rotated to give a trace vector for the slip line (Figure 3b), which is then transformed via a further rotation and the EBSD data (Figure 3c) to a vector in ‘crystal space’ (Figure 3d). This transforms the slip trace from a ‘specimen symmetry’ into one with ‘crystal symmetry’, e.g., [0.5 0.5 0.5] to [110]. If the dot product of the slip plane normal and this vector is 90° ± ∆, where ∆ is a misorientation threshold of ~5° used to account for small experimental misorientations, then the observed surface trace lies within the plane (Figure 3e).

Note that this solution is not unique as several planes within the unit cell may contain the slip trace; Schröders et al. report a total indexing frequency of 172%, for example [16]. However, it is experimentally “cheap”, and is able to guide identification of slip systems for further, more detailed analysis via micropillar compression.

### 2.5. Automated Analysis of Slip Lines

**Figure 4:**
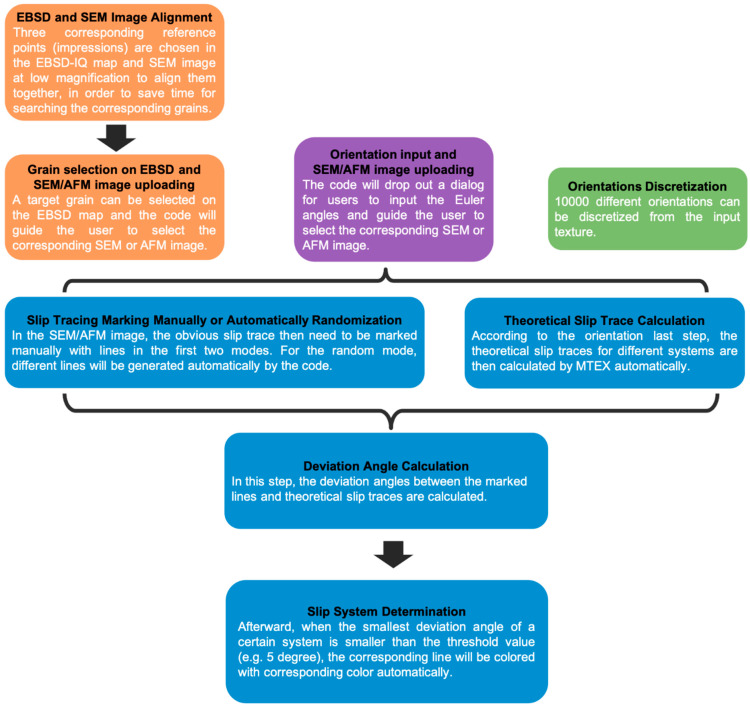
Workflow of the automated slip line analysis code. Orange segments show analysis steps corresponding to the input of EBSD maps (mode 1), purple shows mode 2 and green shows mode 3. All the blue segments then run to give the final assignment of a slip line to a slip system.

Such a series of vector rotations is most easily done via a (semi-)automated analysis. Here, we give an example of MATLAB code (Version 2015 or newer) with MTEX toolbox (Version 5.3.1) developed for this purpose within our group. Figure 4 shows the workflow of the code, and the various options available when automating the slip line analysis. The code is split into three sections, in which the orientation data is entered, the slip lines identified, and the active slip planes determined. For the initial entry of orientation data, a further three modes are available, based upon (i) EBSD maps, most often used in the case of polycrystals, (ii) single orientations where the indents are contained within a single grain, or (iii) where the sample orientation in the indented area has not been directly measured, and a set of orientations should be generated given either an assumed texture or one measured separately by X-ray diffraction (XRD) for example.

#### 2.5.1. Entry of Orientation Data

##### Mode 1: EBSD Maps

This mode is most commonly used where indents are made in a polycrystalline sample, such that the number of orientations to include in the analysis approaches the number of indents made. The code therefore takes EBSD data collected over the area of interest and aligns it to secondary electron (SE) images of the indents to correlate the location data with the orientation data before the user indicates the slip lines of interest and the slip planes are calculated. The EBSD data are typically OSC formatted data, but the code leverages MTEX and is therefore flexible. The specific steps to achieve the slip plane calculation are:

- Step 1: Alignment. If there is misalignment between the SEM image of the indents and the EBSD map, an artificial angle deviation could be introduced between the experimental slip lines and those theoretically possible based on the crystal symmetry slip traces. To avoid this, in this step the code will firstly plot and save the EBSD-IQ map and then guide the user to upload an alignment SE image taken under low magnification. Afterward, the user can select three different indents (e.g., the corners of the array of indents) to use as fiducial markers to align the two images. The code will determine the best fitted alignment parameters based on the selected positions of the indents.
- Step 2: Indent Selection. In this step, a grain of interest on the output IQ image is selected and the corresponding SE/AFM image of the indent to be analysed should be uploaded.

##### Mode 2: Single Orientation

In this mode, no upload of the EBSD data is necessary. Instead, the code will ask for the Euler angles of the orientation and then for the upload of the corresponding SE/AFM image of the indent of interest.

##### Mode 3: Random Case from Texture

In mode 3, the active fraction of slip systems is then calculated only considering the effect of the bulk texture and the Schmidt factor for each slip system. The user must upload the texture of the specimen as an input–typically measured by X-ray diffraction- and the code subsequently discretises this texture into 10,000 orientations. The (nanoindentation) Schmid factor of each slip system is calculated based upon a series of points distributed over the assumed stress field under the indenter [41,42,43]. It is assumed that those with a Schmid factor greater than 0.4 will be activated. This mode, therefore, allows an analysis in the absence of direct orientation measurement; however, it relies on the assumptions that the given texture is representative of the indented region, and that the stress field is homogenous enough to be described by pure theory.

#### 2.5.2. Slip Line Identification

In the first two modes, the code will firstly rotate the SE/AFM image automatically according to the alignment parameters determined in Section 1. The user subsequently needs to mark the slip traces of interest on the rotated image by clicking the start and end points of each of the traces. The traces will be marked with black dashed lines. In mode 3, the code will go through the 10,000 orientations one by one and generate different slip lines (0–180 degrees relative to the x-vector).

Simultaneously, the code can also calculate the theoretical slip lines of different slip planes of interest on the observed surface.

#### 2.5.3. Active Slip Plane Determination

The deviation angle between the user-identified and/or automatically generated lines and the lines that each theoretical slip plane would produce can then be calculated. When this angle is small, i.e., the misorientation threshold, ∆, is under ~5°, it can be assumed that the slip plane of interest is active. This misorientation threshold can be varied according to the needs of the user; larger values will be more forgiving to experimental error, cross slip, slip line curvature, etc, but will result in an increase in over-counting, as each experimental slip trace could correspond to an increased number of potential slip planes.

**Figure 5:**
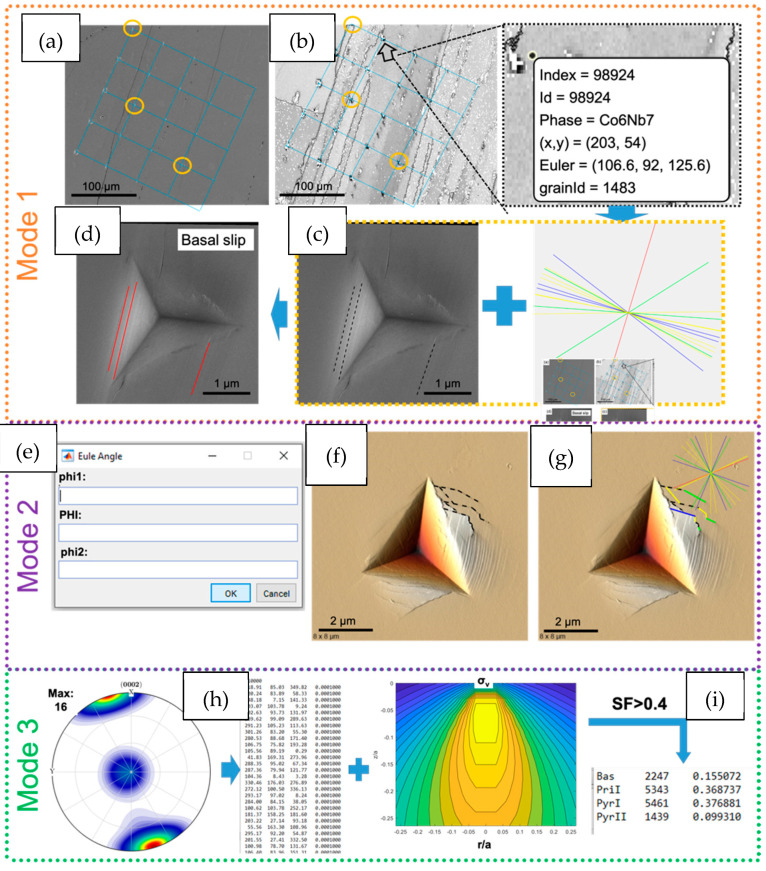
Examples of indents analysed in mode 1, mode 2 and mode 3. (**a**) In mode 1, the three points indicated by the yellow circles are used to align the EBSD and SE images. (**b**) The indent of interest is selected from the EBSQ-IQ map, (**c**) the corresponding SE image is uploaded, and the slip lines are marked by the user. (**d**) The code subsequently identifies the operating slip planes, and colours them accordingly. (**e**) In mode 2, the Euler angles are provided by the user, (**f**) the slip lines are marked by the user on the AFM image, (**g**) and then these are assigned to slip planes and coloured accordingly. (**h**) In mode 3, the global texture of the sample is defined as an input and discritised into 10,000 orientations. (**i**) The stress field under an indent is calculated for these orientations, and slip systems showing a Schmid factor >0.4 are assumed to be active. The indents shown in (**b**–**d**) are made in a Nb_6_Co_7_ μ-phase, produced by arc melting and annealed for 1000 h at 1100 °C and mechanically polished down to a colloidal silica finish. The indents in (**f**,**g**) are in the same Fe_7_Mo_6_ µ-phase sample described in Figure 3.

The MATLAB code operating in modes 1 and 2 will colour the slip traces corresponding to the slip plane operating, e.g., basal planes, pyramidal planes, etc. For mode 3, the activity of this slip plane will simply be counted. An example of these modes are shown in Figure 5.

### 2.6. Statistical Slip Plane Analysis

Armed with the knowledge of the active slip planes, it must then be determined what frequency of slip planes would be observed when all values of critical resolved shear stress (CRSS) are equal. For example, for crystals based upon a hexagonal structure, such as the µ-phase or many of the Laves phases, it would be expected that prismatic slip is observed six times more often than basal slip. This is simply due to the fact that there are six different prismatic planes available for each basal plane. If the sample itself is textured and/or the number of grains sampled does not reflect a random texture, then the local texture of the indented area thus needs to be taken into account. This can be achieved, for example, by taking ~10^2^–10^3^ random slip trace angles and analysing them in an identical way to the “real” data, i.e., with factors such as the misorientation threshold between the slip trace measured experimentally and the slip plane of the crystal or any exclusions of planes running nearly parallel to the surface kept constant.

Deviations from this theoretical, background slip line frequency, which gives the distribution of slip traces expected for equal values of CRSS on each considered slip system, are, therefore, likely representative of differences in the CRSS needed to activate the different slip systems. In this case, deviations towards a higher number of slip traces observed for a given plane would indicate a low CRSS, while high values of CRSS lead to a lower observation frequency.

As another intermetallics example, the hexagonal C14 Laves phase exhibits all the typical slip systems of a hexagonal material: basal, 1st and 2nd order prismatic, and 1st and 2nd order pyramidal slip systems [6]. In Mg_2_Ca, basal and 1st order prismatic slip lines show negligible deviations from the theoretical analysis, and the values of their CRSSs were also found to be similar by microcompression: 0.52 ± 0.05 GPa and 0.53 ± 0.07 GPa, respectively. It is, therefore, probable that, for slip systems that fall into distinct groups of equal frequency compared with the background, it is only necessary to investigate one member via micropillar compression, and the CRSS for all the slip systems in the group may be approximated as similar.

Overall, the slip trace analysis, therefore, yields two important pieces of information: the active slip planes (without slip directions of course) and their likely relative ranking in terms of CRSS.

### 2.7. Verification of Active Slip Planes and Identification of Slip Directions or Burgers Vector

In order to determine which slip systems, not just which slip planes, are operating in the material, further analysis of the dislocation character using the TEM is required. This typically takes place via a **g**·b analysis (where **g** is the incident beam direction and **b** the Burgers vector of the dislocation) and is particularly important in materials where the slip systems are not yet known, as the Schmid factor cannot be calculated until the Burgers vector is known. In this case, TEM must be performed before the Schmid factor can be calculated and values of CRSS are determined.

This analysis is normally done based on indents rather than micropillars, as indents contain regions of low dislocation density and more slip systems in one membrane. TEM of a pillar usually serves to confirm the active slip plane, but the high dislocation density often precludes a Burgers vector analysis due to the high lattice strain affecting visibility in the required two-beam-conditions. A detailed description of the lift-out process is not given here, as there is already a significant body of work on the topic, e.g., [44].

It is important to note, however, that for complex intermetallics, a deformation mechanism beyond simple dislocation glide may be operating, for example, the synchroshear mechanisms on the basal plane of the µ-phase [15] or formation of meta-dislocations [45]. In these cases, a simple identification of the slip plane and Burgers vector are not enough to infer the atomic mechanisms of dislocation glide. Here, HR-TEM may be of help [15,45], but will require careful alignment and further thinning of the sample for the atomic-scale visualisation of defect structures.

### 2.8. Micropillar Milling and Determination of CRSS

Once the necessary slip systems required for further investigation have been identified, they must be isolated in a uniaxial testing environment capable of initiating slip only on the system of interest. As discussed, for hard and brittle materials, this is not possible macroscopically due to the lower bound of critical flaw size restricting practical tests. Therefore, micropillars are typically employed; a review particularly focused on their application to brittle materials is available elsewhere [26].

Micropillars are typically milled with either a square or cylindrical geometry, the choice between which is largely left up to the reader. In copper, there was no observed difference in the yield or work-hardening response of square and round micropillars [46]. Therefore, so long as square pillars do not require significantly more exposure to the focused ion beam—which is well known to affect the mechanical response [47]—then a compromise between ease-of-manufacture, favouring cylindrical pillars, and a potentially easier identification of slip systems for well-aligned square pillars must be decided on for each material. By calculating Schmid factors for the slip systems of interest in sufficiently large grains identified by EBSD, it is possible to produce micropillars in crystal orientations selected to only activate a single slip system. As noted above, where slip systems are not yet known, the Burgers vector must be identified using TEM before the Schmid factor can be calculated. Subsequently, after compression, parallel slip traces at a constant angle to the compression direction are normally observed.

For the compression tests themselves, provided the feedback loop of the nanoindenter employed for such tests is rapid enough (or the indenter is intrinsically controlled in this manner), compression testing is often best performed under ‘constant displacement rate’ testing, as, in our experience, this allows for better control of the final strain. It is common that rapid displacement bursts are seen when slip initiates, and, hence, a reduction in the applied load is necessary to prevent over-compression of the pillar and a subsequent inability to observe clear slip lines.

**Figure 6:**
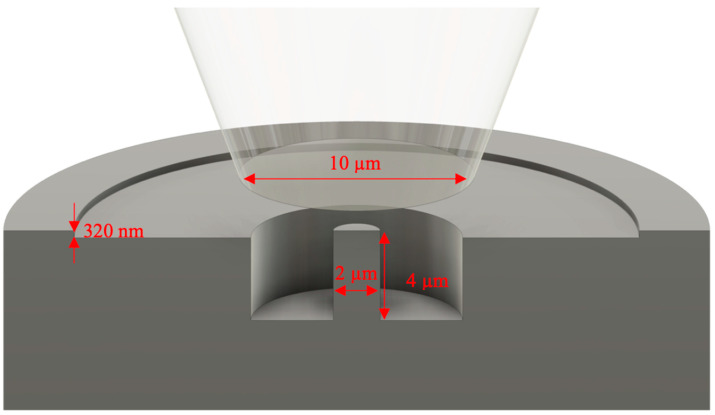
Staircase milling to control of the final pillar strain in the case of strain bursts too large to be reliably controlled using the nanoindenter. The shallow initial cut prevents the flat punch from further compressing the pillar once slip initiates.

In the event that the control loop of the indenter (for a load-controlled machine) does not prove to be rapid enough to prevent this over-compression, the final strain can also be controlled physically by a “staircase” milling procedure. This is shown schematically in Figure 6, along with other typical dimensions. The first FIB cut is only carried out to a depth equal to the final desired strain: e.g., for a pillar that will be 4 µm in height, with a desired final strain of 8%, the first cut is only 4000 × 0.08 = 320 nm in depth. Upon failure of the pillar, this shallow outer region prevents the flat punch from compressing the pillar further. In this setup, the inner, deeper radius must be small enough to give the flat punch a large contact area for efficient deceleration, but large enough to obstruct imaging of the deformed pillar as little as possible. Figure 6 also displays other typical features of a micropillar test, done to facilitate a successful test even with the typical misalignments that occur when moving between an optical microscope and the indenter position. These include a flat punch diameter significantly larger than that of the pillar, and a much larger outer diameter (around 25 µm) so that the punch cannot simultaneously contact the micropillar and the un-milled surface.

In order to identify these misalignments, it is most convenient (if the indenter in question supports it) to monitor an instant calculation of the Young’s modulus of the pillar, based upon the applied force and displacement throughout the test. This needs only to be calculated based upon nominal micropillar dimensions, and without any corrections for a precise strain calculation (a Sneddon correction [42]), as the difference in contact stiffness between a micropillar and the surface of the sample results in Young’s moduli that differ by several orders of magnitude. The researcher can, therefore, be confident that a reported modulus of hundreds of GPa means the pillar is successfully being compressed, while a result in the range of TPa means the pillar has been missed and the test can be aborted without having to wait further.

A final strain of 8% is often a good target: this allows significant slip to take place but usually without too many active systems. It additionally provides a final flow stress representative of that achieved in nanoindentation. However, this naturally depends on the crystal structure of interest, number of potential dislocation sources in the pillar, etc., so must be evaluated each time.

Once slip traces have been produced, they must be correlated with potential slip planes that are possible for the crystal structure of interest. This is not always straightforward, even in relatively simple crystal structures. For example, in BCC materials the possible slip system families, namely {110}, {112} and {123} <111>, show very similar slip traces with respect to the angle that they will make on the surface of the pillar. The shared slip direction precludes further identification using this as a factor, but even if a shared slip direction were not the case, friction between the sliding top of the pillar and the flat punch often interferes with a clear identification of the slip direction.

**Figure 7:**
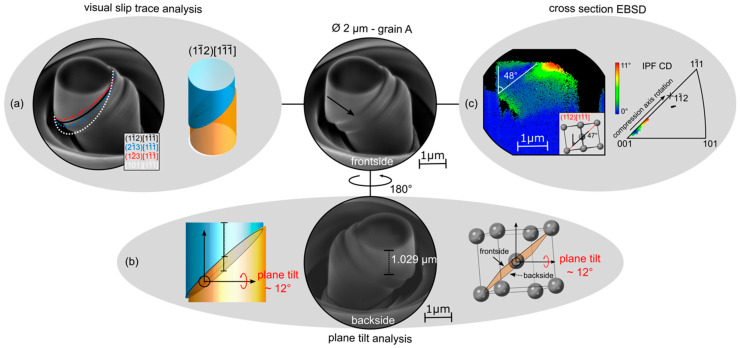
Three different slip system analysis methods demonstrated on a BCC Fe-2.4wt%Si micropillar. (**a**) showing the visual slip trace analysis conducted on a pillar tilted to 45°. This is supported by a cylindrical MATLAB model utilising the toolbox MTEX, (**b**) plane tilt analysis utilising the same MATLAB model and VESTA [49] to visualise the tilt of a slip plane in an edge on view (effectively a 90° tilt) compared to a calculated value from measurements of two SE-images taken from opposite sides, (**c**) cross section EBSD results: misorientation deviation map with indicated slip plane angle as well as IPF triangle relative to the compression direction (CD) with indicated relative rotation of the compression axis towards the (*hkl*) pole of the identified slip plane, the slip plane positions and the corresponding colour codes including scales for the orientation deviation/axis rotation. Image reproduced from [8].

In addition, ongoing deformation can alter the appearance of initial slip traces. With progressive deformation, the slip trace angle can become smaller and less obvious through compression and multiple slip can also occur, either due to friction or from activation of a second slip system. Finally, misalignments between the SE-image and the EBSD measurement can further distort the results. To ensure that the right slip system is found, the results of several analysis methods can be applied, three methods of which are presented in Figure 7, using a single crystal of BCC iron as an example [8], and described in detail below.

#### 2.8.1. Method 1: Visual Slip Trace Analysis

The typical method of correlating slip traces with active slip planes is a simple, direct visual slip trace analysis. Here the SE-image taken post-compression is compared to a theoretical model showing varying slip systems (Figure 7a) created using MATLAB along with the toolbox MTEX [48]. The specific methods for creating this model using these are discussed below in Section 2.9.

The unit cell within the model is oriented according to the EBSD measurement of the micropillar area and is then rotated to match the same view as in the SE image of the micropillar. Furthermore, the model needs to have the same properties as the chosen material: crystal structure, lattice parameter, occurring planes/directions etc., in order to produce a visually comparable slip plane. The top diameter of the cylindrical model is adjusted to match the micropillar, then overlaid on the slipped area. The slip plane displayed in the model that shows the smallest deviation from the observed slip traces is then assumed to be the active slip system.

#### 2.8.2. Method 2: Plane Tilt Analysis

A second approach is the plane tilt analysis, which is essentially a similar analysis to the slip trace analysis but conducted from both sides of the pillar [8]. Two images of the deformed micropillar from opposite sides (a 180° rotation) are required. The vertical distance from the lower apex of the top face to the first slip trace is subsequently measured on either side and corrected for tilt in the SE image. Special care must be taken in the event of multiple slip traces that the same trace is identified and measured each time. These distances and the diameter of the micropillar can be used to calculate the slip plane tilt for an edge on view (i.e., a 90° stage tilt). This can then be compared with theoretical tilts of different slip systems as seen in a unit cell simulator like VESTA (verson 3.5.7 or greater) [50] or in the presented cylindrical MATLAB model (Figure 7b).

#### 2.8.3. Method 3: EBSD Cross-Sectional Analysis

The third approach involves performing EBSD on a cross-section of the micropillar. The pillar is cut out of the macroscopic sample, attached to a TEM grid, and then thinned to the centre from one side. The coarser nature of the technique compared with TEM analysis means low-kV polishing steps are typically not necessary. In the next step an EBSD measurement is performed for which the high angle required for EBSD, e.g., 70° to the electron beam, must be obtainable without shadowing by either holder or TEM grid.

Figure 7c shows an example of the data obtained with this technique. The EBSD data are plotted as relative misorientation compared to the base of the pillar. The slip plane angle can subsequently be measured indirectly using features in the misorientation map; due to the fact that dislocation storage, which is represented by local misorientation, varies from the bulk, and is affected by the presence of slip planes as these store dislocations themselves and often obstruct motion of dislocations on intersecting slip planes, leading to further storage that highlights the position of the most active areas of slip and their orientation. The measured angle can then be compared to slip plane angles in one of the theoretical models (as above, images of the oriented unit cell or the 3D model of the pillar for an edge-on view). As additional information, in a laterally constrained uniaxial compression test, the slip plane normal should rotate towards the compression axis, due to rotation of the crystal. In the inverse pole figure, this is visualised as the crystal direction corresponding to the compression axis ‘moving’ towards the pole of the expected slip plane, that is the trace between the undeformed bottom or bulk region of the map and the deformed region of interest closer to the top of the pillar follows this trajectory.

The combination of these analyses allows a reliable conclusion of the activated slip plane, and, if the respective Burgers vector is already known, also the corresponding slip system. In the example analysis displayed in Figure 7, all three methods indicate that this is $\left(1\bar{1}2\right)\left[1\bar{1}\bar{1}\right]$. In Figure 7a. $\left(1\bar{1}2\right)\left[1\bar{1}\bar{1}\right]$ gives the best fit comparing the 45° micropillar SE-image to the cylindrical MATLAB model, although it can be seen that both $\left(2\bar{1}3\right)\left[1\bar{1}\bar{1}\right]$ and $\left(1\bar{2}3\right)\left[1\bar{1}\bar{1}\right]$ are similar. However, the tilt analysis in Figure 7b matches best that expected for $\left(1\bar{1}2\right)\left[1\bar{1}\bar{1}\right]$, supporting this conclusion. This can be further confirmed by EBSD and the effective rotation of the compression axis towards $\left[1\bar{12}\right]$ (Figure 7c).

### 2.9. Use of MTEX to Produce Pillar Visualisations

The incorporation of MTEX 5.1.1 in the micropillar MATLAB model allows all the standard slip systems to be easily selected, as well as providing a framework to navigate within Euler space. Similarly, unexpected or deviating slip systems can also be easily incorporated into the code. The current implementation allows a simple preselection of slip systems suitable for the imported cif file (Crystallographic Information File) (e.g., BCC FeSi). The required inputs are:

- The cif file;
- The measured pillar orientation (Euler angle);
- The displayed slip system for comparison with the experimental slip lines;
- The rotation of the cylinder to match the micropillar side view SE-image, as well as the offset direction and magnitude.

This produces a model with top, 45° and 90° views of a cylindric micropillar model with slip along the selected slip plane/direction. With this, one can compare the experimental SE pictures with the cylindrical model to find the slip system with minimal deviation from experimental slip results, as shown in Figure 7a.

## 3. Discussion

The methods discussed above are available in many labs but are less commonly combined for the systematic study of deformation in hard crystals. Once all the methods are mastered, the investigations themselves are fairly straightforward, and until now have largely, in our opinion, been simply limited by the amount of personnel time required for each step. By improved automation, in part with the tools presented here, this requirement is significantly reduced, allowing the researcher to instead conduct improved experiments. For example, using more indents or more micropillars to achieve a better statistical understanding of their samples, or being able to ‘scan’ over finer compositional changes to elucidate the effect of alloying elements.

Regardless of the application, however, the limitations of small-scale testing must be borne in mind. Surface effects will influence or dominate dislocation nucleation, particularly in micropillars. Small indents and pillars are subject to the ‘smaller is stronger’ size effect, affecting the direct transferability of measured hardness or critical stresses. The high strains underneath a Berkovich indenter can affect cross-slip, particularly in anisotropic materials, further affecting the measured trends as well as absolute values [50]. The reader is therefore strongly encouraged to also refer to the reviews published in key areas, such as microcompression [22,26], elevated temperature testing [51,52], or hexagonal materials [53].

All the codes discussed in this work are maintained in an online repository available at the following reference: [54]. 

## 4. Conclusions

Whether studying the plasticity of individual grains and grain boundaries, or the multitude of intermetallic phases available, the deformation mechanisms of crystal structures are of enormous importance. These studies, however, are often bottlenecked by the need to perform many steps manually, or for automation tools to be developed again and again in each lab. We have, therefore, provided a tutorial of the tools developed at IMM, taking the reader through all necessary steps to collect, analyse and interpret data on plasticity in hard crystals. Nanoindentation combined with orientation measurements and the code given here allows rapid, statistical analysis of active slip planes in order to guide microcompression experiments. These compression tests allow isolated slip on the plane of interest. With the supplied experimental “tricks”, plastic deformation is possible without initiating brittle fracture while ensuring the analysis of the slip lines is simple yet robustly accurate.

## Data Availability

All the codes discussed in this work are maintained in an online repository available at https://git.rwth-aachen.de/Sandra.Korte.Kerzel/slip_system_analysis_tools/.
